# Improving quality and safety of care in nursing homes by team support for strengths use: A survey study

**DOI:** 10.1371/journal.pone.0200065

**Published:** 2018-07-02

**Authors:** Martina Buljac-Samardžić, Marianne van Woerkom

**Affiliations:** 1 Erasmus University Rotterdam, Erasmus School of Health Policy & Management, Rotterdam, The Netherlands; 2 University of Tilburg, Department of Human Resource Studies, Tilburg, The Netherlands; Brown University, UNITED STATES

## Abstract

Growing evidence suggests that workload has an adverse effect on quality of care and patient safety in nursing homes. A novel job resource that may improve quality of care and patient safety and alleviate the negative effect of workload in nursing homes is *team support for strengths use*. This refers to team members’ beliefs concerning the extent to which the team they work in actively supports them in applying their individual strengths at work. The objective was to investigate the relationships between workload, team support for strengths use, quality of care, and patient safety in nursing homes. We collected (cross-sectional) survey data from 497 caregivers from 74 teams in seven different nursing homes. The survey included measures on perceived workload, team support for strengths use, caregivers’ perception of the quality of care provided by the team and four safety incidents (i.e. fall incidents, medication errors, pressure ulcers, incidents of aggression). After controlling for age, team size, team tenure, organizational tenure, and nursing home, multilevel regression analyses (i.e. individual and team level) showed that perceived workload was not significantly related to perceived team-based quality of care and the frequency of safety incidents. Team support for strengths use was positively related to perceived team-based quality of care, negatively related to medication errors, but not significantly related to fall incidents, pressure ulcers, and aggression incidents. Finally, we found that perceived workload had a negative effect on perceived team-based quality of care when team support for strengths use is low and no significant effect on perceived team-based quality of care when team support for strengths use is high. This study provides promising evidence for a novel avenue for promoting team-based quality of care in nursing homes.

## Introduction

Concerns about workload in nursing homes have increased in the past few decades. Nursing homes deal with an increasing workload due to the rising number of elderly people, financial difficulties, understaffing, increased complexity of care, and higher expectations regarding the quality of care [1]. Excessive workload is associated with undesirable outcomes and is internationally recognized as an urgent topic [2–9]. Scholaski et al. [10] showed that workload has a negative effect on nurse-perceived quality of care and patient safety and several studies show that the workload of nurses has an adverse effect on patient safety issues such as infections [3], patient falls,[4] medication errors [4], and patient mortality [2, 5, 6]. Because caregivers interact intensively with the patient, they are able to assess the patient’s condition and listen to any concerns that the patient may voice [11]. However, when confronted with a high workload caregivers may not have the time to assess the psychosocial and physical status of patients due to limited opportunities to interact with patients and other caregivers [11, 12]. This may hinder the proactive care that detects early signs of clinical deterioration or complications and arranges follow-up interventions, resulting in leaving at least one essential task undone [11, 12]. Consequently, quality of care and patient safety will be diminished [11–14].

### Team support for strengths use

Job resources refer to the physical, psychological, social, or organizational aspects of the job that facilitate the achievement of work goals and counteract the strain associated with job demands [15, 16]. In this paper, we argue that *team support for strengths use* is a novel job resource that may improve the quality and safety of care in nursing homes. It refers to team members’ beliefs concerning the extent to which the team they work in actively supports them in applying their individual strengths at work [17, 18]. Individual strengths are trait-like abilities that potentially allow a person to perform at their personal best [19]. When applied, strengths are personal assets that can buffer against stressors and help a person to overcome setbacks [20]. For instance, a nurse with a good sense of humor can make use of this strength to calm a patient in distress. Recent studies have shown that support for the use of strengths is a resource that facilitates job performance and alleviates the negative effects of job demands [21, 22].

In nursing homes, the team is an important contextual factor [23] that may influence whether caregivers are able to leverage their strengths. If team members know what strengths their co-workers have and tasks are allocated in line with their unique qualities, caregivers might be better able to leverage their strengths at work [21, 22]. For instance, in a nursing home team, nurses may stimulate self-management by training patients in the necessary skills or by their interactions with individual patients during daily activities. When a team offers strengths-use support, a nurse whose strength is social intelligence could choose to enhance patient self-management by engaging in social interaction, potentially freeing other team members to enhance self-management in ways that better suit their own strengths.

Employees who feel supported to use their strengths can be more authentic [24], resulting in lower levels of stress and higher levels of energy and coping skills [25]. Therefore, caregivers who work in a team that offers strengths-use support may experience fewer error-inducing conditions such as stress and fatigue which may lead to a higher quality and safety of care delivery [24–26]. Moreover, these caregivers are more likely to appreciate their colleagues for their strengths, thereby potentially creating a blame-free environment in which caregivers are willing to report errors and develop strategies to improve the quality and safety of care [26].

### The moderating role of team support for strengths use

Baethge, Muller, and Rigotti show that nurses who focus their resources on specific goals as opposed to spreading them across multiple goals are better able to maintain performance while facing a high workload [27]. In line with this principle, we also expect that caregivers who can capitalize on their strengths, instead of having to be a jack of all trades, can better maintain the quality and safety of care when confronted with a high workload. These caregivers experience higher levels of self-esteem because they feel valued for their unique qualities that they bring to the team [19]. In turn, enhanced self-esteem compensates for the threats to self-esteem that occur in response to the appraisal of stress resulting from a high workload, making caregivers more effective in coping with high job demands [28]. Furthermore, emotional or distressing situations cause less stress when caregivers can approach these situations from their own strengths. In the previous example of the nurse, enhancing patient self-management in addition to other tasks may add to the workload, but it will be less stressful if the nurse can choose to deal with this task in line with his/her individual strengths. Using team support for strengths use as a coping strategy helps caregivers deal with high workload as it creates the ability to alleviate stress [29]. Buffering or reducing the stress level of caregivers within a team is generally seen as an effective way to reduce safety errors and improve the quality of care provided by the team [29].

### Aim

The aim of this study was to investigate the relationships between perceived workload, team support for strengths use, the perception of the quality of care provided by the team, and the four most frequently registered indicators of patient safety in Dutch nursing homes, namely fall incidents, medication errors, pressure ulcers, and aggression incidents [29].

Hypothesis 1: Perceived workload is negatively related to (a) perceived team-based quality of care and positively related to perceived frequency of (b) fall incidents, (c) medication errors, (d) pressure ulcers, and (e) aggression incidents.

Hypothesis 2: Team support for strengths use is positively related to (a) perceived patient safety and negatively related to perceived frequency of (b) fall incidents, (c) medication errors, (d) pressure ulcers, and (e) aggression incidents.

Hypothesis 3: Team support for strengths use weakens the relationship between perceived workload on the one hand and perceived (a) team-based quality of care, (b) fall incidents, (c) medication errors, (d) pressure ulcers, and (e) aggression incidents on the other hand.

## Methods

### Field of study

The way in which nursing homes are organized and financed differs per country. For instance, Dutch nursing homes are relatively large, with an average of approximately 189 beds in comparison to an average of 107 beds in US nursing homes. In addition, the majority of caregivers in Dutch nursing homes are licensed practical nurses, whereas in US nursing homes certified aides form the majority of the staff. Registered nurses are more common in US nursing homes in comparison to Dutch nursing homes [30–33]. However, in spite of these differences, in most developed countries with high life expectancy (such as the Netherlands, Belgium, Denmark, Germany, and US), nursing homes face similar issues that increase the workload, such as an increase in number of elderly people (with multiple chronic diseases), financial restrictions, understaffing, and increased quality expectations [33, 34]. As a response to those issues, a major trend in Europe is the marketization and privatization of nursing homes, which is similar to the well-established pattern in the US. Nevertheless, currently the majority of Dutch nursing homes are non-profit organizations, whereas for-profit nursing homes form the majority of US nursing home [33, 34].

### Sample

For this study ‘Strengthening Patient Safety in Nursing Homes’ (i.e. SPS4NH) we conducted a cross-sectional survey amongst nursing homes in the Netherlands that were part of our personal network. Seven nursing homes agreed to participate in a cross-sectional survey with several teams of nurse aides, registered nurses, and licensed practical nurses. In some cases, occupational, speech, and physical therapists were also members of the team. We asked the HR director and/ or the unit managers of the nursing homes to select participating teams that were representative for their whole organization in terms of type of patients and type of teams. The nursing homes were located across the Netherlands and their size ranged from small (800 employees) to large (5500 employees).

In 2015 data were collected among 1116 employees in 84 teams that provide direct care to clients. The researchers sent these participants a questionnaire with a cover letter that introduced the aim of the project and ensured their anonymity. After two weeks the respondents were sent a reminder to complete the survey. In total, 497 respondents (74 teams within 7 nursing homes) completed the questionnaire, resulting in a response rate of 44.5%. Team size varied from 5–40 team members with a mean of 16.96 members (SD = 8.79).

### Measures

We measured *perceived workload* with an eight-item scale developed by De Jonge et al. [35]. This scale has been validated in several studies, with Cronbach’s alpha’s varying between .86 and .89 [36–40]. In line with other researchers [e.g. 27, 37, 38, 41–44], we chose to measure perceived workload instead of objective workload because caregivers may perceive the same workload differently dependent on their employment status, career goals and expectations, coping mechanisms, and work ethics [44, 45]. Moreover, indicators of actual workload only take into account quantitative aspects, whereas the scale by De Jonge et al. takes both quantitative and qualitative aspects of workload into account. An example item is: “In my team, too much work needs to be done”. In this study, perceived workload had a Cronbach’s alpha of .87.

*Team support for strengths use* was based upon a validated scale [46, 47], but was adapted to refer to the team context. We investigated the validity of this adapted seven-item scale with the following procedure. First, we made a random split of our dataset so that we could conduct an Exploratory Factor Analysis (EFA) on one half of the dataset (N = 228), and a Confirmatory Factor Analysis (CFA) on the other half of the data (N = 269). Results of the EFA indicated that the seven items loaded on one factor, with an Eigenvalue of 3.59, explaining 51.31% of the variance. Second, we conducted a Confirmatory Factor Analysis (CFA) on the other half of the data (N = 269). Results of the CFA indicated that this one factor model had an acceptable fit to the data (*χ*2(14) = 63.17, CFI = .92, SRMR = .05). An example item is “In this team, my tasks are adjusted to suit my strengths”. The Cronbach’s alpha of team support for strengths use was .84.

In this study quality of care refers to caregivers’ perception of the quality of care provided by the team and is therefore labelled as *perceived team-based quality of care*. Caregivers have an ideal position for assessing the quality of care that is provided by the team as they build their perception overtime on various encounters and processes, based on interactions with fellow caregivers, informal caregivers, and patients [48, 49]. Moreover, previous studies found that nurse-reported quality of care is associated with objective outcomes such as hospital mortality rates, 30 day inpatient mortality, 30 day failure to rescue, and survival probabilities [48, 50]. Perceived team-based quality of care was measured by a self-developed scale, which consisted of five items. “The way our team works guarantees a good quality of care” is an example item. We applied the same validation procedure as for the strengths-based team support scale. Results of the EFA indicated that the five items loaded on one factor, with an Eigenvalue of 2.62, explaining 52.83% of the variance. Results of the CFA showed that this one factor model had a good fit to the data (*χ*2(5) = 14.33, CFI = .97, SRMR = .03). Perceived quality of care had a Cronbach’s alpha of .75.

In addition, we performed a CFA that included perceived workload, strengths-based team support and perceived team-based quality of care. A CFA three-factor model with all three scales loading on three separate factors (*χ*2(167) = 346.36, CFI = .91, SRMR = .07,) fitted significantly better to the data than a two-factor model with perceived workload and quality of care loading on one factor and team support for strengths use loading on a second (∆*χ*2(2) = 338.08, *p* < .001; CFI = .74, SRMR = .13), a model with team support for strengths use and quality of care loading on one factor and perceived workload loading on a second factor (∆*χ*2(2) = 168.24, *p* < .001; CFI = .83, SRMR = .08), and a model with all three scales loading on one common factor (∆*χ*2(3) 806.91, *p* < .001; CFI = .51, SRMR = .18.

Perceived workload, team support for strengths use, and perceived quality of care were all measured with five-point Likert scales ranging from 1 (strongly disagree) to 5 (strongly agree).

We measured *perceived patient safety* by asking respondents to rate four safety indicators, namely the frequency of fall incidents, medication errors, pressure ulcers, and aggression incidents on a five-point Likert scale from (1) never to (5) often.

Gender, age, education, number of team members, team tenure, organizational tenure, and job title were included as *control variables*. All measures that were included in this study are presented in S1 File.

### Data analysis

The analyses were conducted using SPSS (Statistical Package for Social Science) 15.0. Hypotheses were tested with multilevel regression analyses using the linear mixed-effects model procedure in SPSS [51] such that the effects of the individual-level variables were examined while accounting for the non-independence of observations within groups [52]. We computed deviance scores (differences in the -2 log likelihood) to compare the different models and to test their significance [53]. Measures of model fit for all models were obtained by comparing deviance scores using a Chi-squared distribution table.

### Ethical consideration

The ethic committee confirmed that this study fell outside the scope of the Netherlands' Medical Research Involving Human Subjects Act (WMO) and therefore no formal ethical approval was needed. Although our research was conducted in a medical setting, it met none of the WMO criteria (http://www.ccmo.nl/en/your-research-does-it-fall-under-the-wmo). First, no patients were involved. Second, the study content and methodology did not constitute an infringement of the physical and/or psychological integrity of the participants. Survey questions on patient safety referred to the team level and were therefore not traceable to individual patients.

## Results

### Sample characteristics

Most of our respondents were female (95.5%), which is slightly more than the percentage of female employees in the Dutch long-term care setting (85%) [54]. The average age of the respondents was 41.06 years (SD = 12.35), and ranged from 16 to 65 years. This is comparable to the general Dutch long-term care sector where the average age of employees is 40.10 years [54]. In comparison with the distribution in Dutch nursing homes our sample included fewer practical nurses (47.4%, compared to 77% in the Dutch population), more nurse aides (21.4%, compared to 11% in the Dutch population), and more registered nurses (15.0%, compared to 7% in the Dutch population), as shown in Table 1 [55]. Respondents had an average team tenure of 4.54 years (SD = 4.51) and an average organizational tenure of 11.42 years (SD = 8.74).

**Table 1:** Characteristics of respondents.

Characteristics	*n* (%)
**Gender** (6 missing values) Female	469 (95.5)
**Education** (12 missing values)	
Secondary degree	102 (21.0)
Vocational degree	349 (72.0)
Bachelor degree	30 (6.2)
Master degree	4 (0.8)
**Job title** (24 missing values)	
Aid	101 (21.4)
Practical nurse	224 (47.4)
Registered nurse	71 (15.0)
Occupational therapist	11 (2.3)
Student nurse	37 (7.8)
Paramedic support	2 (0.4)
Coordinator	27 (5.7)

Descriptive statistics of the main variables are presented in Table 2. The majority of respondents (74.5%) perceived their workload as problematic (i.e. score between 3 and 5 on a 5 point Likert scale) and 22.1% of the respondents perceived their workload as very high (i.e. score between 4 and 5 on a 5 point Likert scale). Most respondents (68.2%) perceived some support (i.e. a score between 3 and 4), and 17.2% experienced strong support for individual strength-use within their team (i.e. a score between 4 and 5). Quality of care provided by the team was considered a problem by 13.3% of the respondents (i.e. score between 1 and 3), whereas 13.4% of the respondents considered the team to provide care of high quality (i.e. score between 4 and 5 score). 22.3% of respondents experienced aggression incidents frequently (i.e. score between 4 and 5), whereas fall incidents, medication errors and pressure ulcers were frequently experienced by only 7.7%, 4.9%, and 0.4% of the respondents, respectively.

**Table 2:** Descriptive statistics.

	**Mean**	***SD***	**% score 1–2**	**% score 2–3**	**% score 3–4**	**% score 4–5**	
Perceived workload*	3.50	.71	1.9	23.6	52.4	22.1	
Team support for strengths use*	3.60	.58	2.1	12.5	68.2	17.2	
Perceived team-based quality of care*	3.63	.52	1.1	12.2	73.3	13.4	
	**Mean**	**SD**	**% score 1**	**% score 2**	**% score 3**	**% score 4**	**% score 5**
Fall incidents**	3.11	.91	1.7	24.2	43.6	22.8	7.7
Medication error**	2.94	.89	2.9	28.9	44.2	19.2	4.9
Pressure ulcers**	2.51	.80	8.0	43.8	37.9	9.9	0.4
Incidents of aggression**	3.65	1.00	1.4	11.6	30.0	34.7	22.3

*score on a 5 point Likert scale ranging from 1 (strongly disagree) to 5 (strongly agree)

** score on a 5 point Likert scale ranging from 1 (never) to 5 (often)

### Correlations

As can be seen in the correlation matrix (Table 3), perceived workload was negatively related to perceived team-based quality of care (*r* = -.12, *p* < .05), and positively related to perceived frequency of fall incidents (*r* = .19, *p* < .01), medication errors (*r* = .19, *p* < .01), pressure ulcers (*r* = .16, *p* < .01), and incidents of aggression (*r* = .13, *p* < .01). Team support for strengths use was positively related to perceived team-based quality of care (*r* = .41, *p* < .01) and negatively related to fall incidents (*r* = -.12, *p* < .05), medication errors (*r* = -.12, *p* < .05), and pressure ulcers (*r* = -.12, *p* < .05). Team support for strengths use did not relate significantly to aggression incidents (*r* = -.05, ns). Perceived team-based quality of care was negatively related to safety in terms of fall incidents (*r* = .24, *p* < .01), medication errors (*r* = .32, *p* < .01), pressure ulcers (*r* = .20, *p* < .01), and aggression incidents (*r* = .18, *p* < .01).

**Table 3:** Correlation matrix.

	Mean	*SD*	MV	1	2	3	4	5	6	7	8	9	10	11	12	13
1. Perceived workload	3.50	.71	18	1												
2. Team Support for Strengths use	3.60	.58	10	-.13**	1											
3. Perceived team-based quality of care	3.64	.53	21	-.12*	.41**	1										
4. Fall incidents	3.11	.91	18	.19**	-.12*	-.24**	1									
5. Medication errors	2.94	.89	44	.19**	-.12*	-.32**	.21**	1								
6. Pressure ulcers	2.51	.80	33	.16**	-.12*	-.20**	.16**	.22**	1							
7. Incidents of aggression	3.65	1.00	13	.13**	-.05	-.18**	.35**	.13**	.12*	1						
8. Gender (1 = man, 2 = female)	1.96	.21	6	.05	.07	.05	-.08	.02	-.05	-.05	1					
9. Age	41.06	12.35	35	.21**	-.03	-.05	.05	.00	-.07	.01	.03	1				
10. Education	4.33	1.69	12	-.06	-.07	-.03	-.01	.09	-.03	.03	.03	-.24**	1			
11. Team size	16.96	8.79	0	.24**	-.04	-.09	.39**	.18**	.16**	.21**	.01	.04	.00	1		
12. Team tenure	4.54	4.51	37	.19**	-.04	.04	.08	.09	.11*	-.02	.01	.32**	-.17**	.17**	1	
13. Organizational tenure	11.42	8.74	26	.10*	.06	.03	-.02	.05	.02	.01	.03	.50**	-.24**	.07	.38**	1
14. Nursing homes:			0													
1 (n = 81)				-.22**	-.13**	-.07	-.20**	-.26**	.01	-.05	-.04	.01	-.05	-.54**	-.01	.01
2 (n = 49)				-.09*	.01	.12**	-.02	.10*	.07	-.07	.01	-.06	-.02	-.05	-.06	-.03
3 (n = 119)				.00	.05	.15**	-.17**	-.15**	-.19*	-.17**	.00	.04	-.02	-.17**	.02	-.05
4 (n = 54)				.01	-.11*	-.19**	-.00	.03	.08	-.00	.03	-.05	.08	.09	-.03	-.01
5 (n = 82)				.12**	.12**	-.03	-.05	.09	-.10*	.27**	-.04	-.04	.06	-.07	-.17**	.00
6 (n = 12)				-.02	.05	-.03	.11*	.28**	.06	-.19**	.01	-.06	-.02	.02	.01	-.04
7 (n = 100)				.17**	-.06	-.07	.32**	.01	.16**	.17**	.04	.09*	.01	.80**	.19**	.09*

**significant at .01 level (2-tailed)

*significant at .05 level (2-tailed)

MV = missing values

Perceived workload was positively related to age (*r* = .21, *p* < .01), team size (*r* = .24, *p* < .01), team tenure (*r* = .19, *p* < .01), and organizational tenure (*r* = .10, *p* < .05). Team size was positively related to fall incidents (*r* = .39, *p* < .01), medication errors (*r* = .18, *p* < .01), pressure ulcers (*r* = .16, *p* < .01), and aggression incidents (*r* = .21, *p* < .01). Team tenure was positively related to pressure ulcers (*r* = .11, *p* < .05). Perceived team-based quality of care was significantly higher in two nursing homes and significantly lower in another nursing home. The same holds for perceived workload and perceived frequency of safety incidents; two nursing homes showed a significantly higher level of perceived workload and more perceived safety incidents, while two other nursing homes showed a significantly lower level of perceived workload and fewer perceived safety incidents. Therefore, dummy variables were included for each of the nursing homes as control variables in further analyses.

### Multilevel analyses

Multilevel analyses are presented in Tables 4–6. All variables were centered before they were entered in the analyses (except for the dummy variables representing the organizations). For each of the outcome variables, model A includes only the control variables. In model B, perceived workload and team support for strengths use were added as main effects while model C also includes the interaction between perceived workload and team support for strengths use. Tables 4–6 shows that perceived workload was not significantly related to perceived team-based quality of care (*B* = -.03, *CI* = -.10, .04) or any of the safety indicators (*B* = .06, *CI* = -.05, .18; *B* = .05 *CI* = -.07, .17; *B* = .07 *CI* = -.04, .18; *B* = .06 *CI* = -.08, .19, respectively), thereby not providing support for Hypothesis 1. Team support for strengths use was positively related to perceived team-based quality of care (*B* = .38, *CI* = .30, .46), negatively related to medication errors (*B* = -.18, *CI* = -.31, -.05), and not significantly related to fall incidents, pressure ulcers, or aggression incidents (*B* = -.11, *CI* = -.24, .02; *B* = -.08, *CI* = -.20, .04; *B* = -.05, *CI* = -.20, .10 respectively), thereby only providing support for Hypotheses 2a and 2c.

**Table 4:** Two-level multilevel regression analysis: Perceived team-based quality of care and frequency of fall incidents.

	Perceived team-based quality of care						Frequency of fall incidents					
	Model 1		Model 2		Model 3		Model 1		Model 2		Model 3	
	Estimate [C.I.]	*p*	Estimate [C.I.]	*p*	Estimate [C.I.]	*p*	Estimate [C.I.]	*p*	Estimate [C.I.]	*p*	Estimate [C.I.]	*p*
Intercept	3.76[3.63,3.90]	.000	3.72 [3.61,3.84]	.000	3.73 [3.62, 3.84]	.000	2.83 [2.57, 3.10]	.000	2.85[2.58, 3.11]	.000	2.84 [2.58, 3.11]	.000
Age	-.00 [-.01,.00]	.185	-.00 [-.01,.00]	.514	-.00 [-.01, .00]	.643	.00 [-.00, .01]	.172	.00 [-.00, .01]	.424	.00 [-.00, .00]	.493
Team size	-.02 [-.04,-.00]	.030	-.01 [-.03,.-00]	.049	-.01 [-.02, -.00]	.032	.04 [.00, .07]	.028	.04 [.00, .07]	.035	.04 [.00, .07]	.033
Team tenure	.00 [-.01,.02]	.655	.00 [-.01,.01]	.566	.00 [-.01, .01]	.635	.01 [-.01, .03]	.354	.00 [-.02, .02]	.707	.00 [-.01, .02]	.672
Organizational tenure	.00 [-.00,.01]	.158	.00 [-.00,.01]	.360	.00 [-.00, .01]	.369	-.00 [-.01, .01]	.367	-.00 [-.01, .01]	.559	-.00 [.01, .01]	.575
Nursing home 1Nursing home 2Nursing home 4Nursing home 5Nursing home 6Nursing home 7	-.43 [-.67,-.19].05 [-.20,.30]-.91 [-1.29,-.52]-.20 [-.41,.01]-.16 [-.38,.06] .05 [-.28,.39]	.001.679.000.062.147.763	-.26 [-.45,-.06].06 [-.14,.25]-.70 [-1.02,-.38]-.22 [-.39,-.06]-.15 [-.32,.03].05 [-.20, .31]	.011.550.000.009.097.685	-.27 [-.46, -.08].07 [-.12, .26]-.67 [-.99, -.36]-.24 [-.41, -.08]-.15 [-.32, .03].07 [-.18, .33]	.007.492.000.004.096.563	.30 [-.15, .75].27 [-.21, .74].49 [-.21, 1.19].17 [-.24, .59].50 [.08, .92].35 [-.33, 1.02]	.196.270.168.409.021.313	.30 [-.16, .76].24 [-.23, .72].42 [-.28, 1.12].16 [-.25, .58].50 [.08, .92].35 [-.32, 1.02]	.194.312.234.435.021.301	.31 [-.15, .77].24 [-.24, .72].40 [-.30, 1.11].18 [-.24, 60].50 [.07, .92].34 [-.34, 1.02]	.184.326.261.393.022.323
Perceived workloadTeam Support for Strengths use Interaction Perceived workload* TSS			-.03 [-.10, .04].38 [.30, .46]	.451.000	-.04 [-.11, .03].36 [.28, .44].14 [.03, .25]	.279.000.013			.06 [-.05, .18]-.11 [-.24, .02]	.243.085	.08 [-.04, .20]-.10 [-.23, .03]-.11 [-.29, .06]	.175.148.192
Teamlevel (intercept)	.03 [.01,.06]	.024	.01 [.00, .05]	.36	.01 [.00, .06]	.40	.18 [.11, .28]	.000	.18 [.11, .28]	.000	.18 [.11, .28]	.000
-2 Log Likelihood	613.99		502.85		496.64		947.42		900.80		899.10	
Deviance change (Δ*χ*2 (df))	-		111.14 (2)**		6.21 (1)*		-		46.62 (2)**		1.70 (1)	
AIC	639.99		532.85		528.64		973.42		930.80		931.10	
BIC	692.48		592.65		592.42		1026.12		990.82		995.12	

**Table 5:** Two-level multilevel regression analysis: Perceived frequency of medication errors and pressure ulcers.

	Frequency of medication errors						Frequency of pressure ulcers					
	Model 1		Model 2		Model 3		Model 1		Model 2		Model 3	
	Estimate [C.I.]	*p*	Estimate [C.I.]	*p*	Estimate [C.I.]	*P*	Estimate [C.I.]	*p*	Estimate [C.I.]	*p*	Estimate [C.I.]	*p*
Intercept	2.77 [2.52, 3.02]	.000	2.74 [2.49, 2.98]	.000	2.73 [2.49, 2.98]	.000	2.36 [2.11, 2.60]	.000	2.36 [2.11, 2.60]	.000	2.35 [2.10, 2.60]	.000
Age	.00 [-.00, .01]	.513	.00 [-.01, .01]	.752	.00 [-.01, .01]	.816	-.00 [-.01, .00]	.102	-.01 [-.01, .00]	.069	-.01 [-.01, .00]	.051
Team size	.01 [-.02, .04]	.436	.01 [-.02, .04]	.625	.01 [-.02, .04]	.596	.01 [-.02, .04]	.519	.01 [-.02, .04]	.641	.01 [-.02, .04]	.605
Team tenure	.01 [-.00, .03]	.161	.01 [-.01, .03]	.259	.01 [-.01, .03]	.252	.02 [.00, .04]	.041	.02 [-.00, .03]	.073	.02 [-.00, .04]	.067
Organizational tenure	.00 [-.01, .01]	.738	.00 [-.01, .01]	.423	.00 [-.01, .01]	.409	-.00 [-.01, .01]	.487	-.00 [-.01, .01]	.504	-.00 [-.01, .01]	.524
Nursing home 1Nursing home 2Nursing home 4Nursing home 5Nursing home 6Nursing home 7	-.16 [-.60, .28].42 [-.05, .88].38 [-.30, 1.06].40 [.00, .80].86 [.45, 1.26]-.02 [-.67, .63]	.463.080.275.049.000.954	-.21 [-.64, .22].41 [-.05, .86].32 [-.34, .98].44 [.05, .82].91 [.51, 1.30].05 [-.57, .67]	.335.079.339.027.000.869	-.20 [-.64, .23].40 [-.05, .86].30 [-.36, .97].45 [.06, .83].90 [.51, 1.30].04 [-.58, .66]	.347.081.362.024.000.904	.28 [-.15, .70].20 [-.24, .64].61 [-.04, 1.27]-.04 [-.42, .35].23 [-.16, .62].22 [-.40, .85]	.197.373.067.849.251.472	.25 [-.19, .68].16 [-.29, .61].57 [-.09, 1.23]-.02 [-.41, .37].24[-.15, .64].26 [-.37, .89]	.261.484.090.902.228.418	.25 [-.18, .69].15 [-.30, .60].54 [-.12, 1.21]-.01 [-.40, .38].24 [-.16, .64].24 [-.39, .87]	.246.507.106.958.236.454
Perceived workload			.05 [-.07, .17]	.396	-.06 [-.06, .18]	.334			.07 [-.04, .18]	.200	.08 [-.03, .19]	.143
Team Support for Strengths use Interaction Perceived workload*TSS			-.18 [-.31, -.05]	.008	-.17 [-.30, -.04]-.08 [-.26, .10]	.013.384			-.08 [-.20, .04]	.193	-.07 [-.19, .06]-.12 [-.28, .05]	.287.158
Teamlevel (intercept)	.15[.09, .26]	.000	.13 [.08, .24]	.000	.13 [.08, .23]	.000	.14 [.08, .25]	.000	.15 [.09, .26]	.000	.15 [.09, .26]	.000
-2 Log Likelihood	912.56		863.45		862.70		888.34		833.94		831.94	
Deviance change (Δ*χ*2 (df))	-		49.11 (2)**		0.75 (1)		-		54.40 (2)**		2.00 (1)	
AIC	938.56		893.45		894.70		914.34		863.94		863.94	
BIC	990.51		952.75		957.95		996.58		923.58		927.56	

**Table 6:** Two-level multilevel regression analysis: Perceived frequency of incidents of aggression.

	Frequency of incidents of aggression					
	Model 1		Model 2		Model 3	
	Estimate [C.I.]	*p*	Estimate [C.I.]	*p*	Estimate [C.I.]	*p*
Intercept	3.47 [3.18, 3.76]	.000	3.46 [3.17, 3.76]	.000	3.46 [3.16, 3.75]	.000
Age	-.00 [-.01, .01]	.770	-.00 [-.01, .01]	.561	-.00 [-.01, .01]	.487
Team size	.05 [.01, .09]	.008	.05 [.01, .09]	.007	.05 [.02, .09]	.006
Team tenure	.01 [-.02, .03]	.627	.00 [-.02, .02]	.957	.00 [-.02, .02]	.900
Organizational tenure	-.00 [-.01, .01]	.928	.00 [-.01, .01]	.678	.00 [-.01, .01]	.657
Nursing home 1Nursing home 2Nursing home 4Nursing home 5Nursing home 6Nursing home 7	.70 [.19, 1.20]-.29 [-.83, .24].40 [-.39, 1.19].80 [.34, 1.26]-.38 [-.86, .09]-.22 [-.96, .53]	.007.276.321.001.111.560	.68 [.17, 1.19]-.32 [-.86, .21].39 [-.40, 1.18].81 [.34, 1.27]-.37 [-.85, .10]-.20 [-.95, .54]	.010.229.330.001.124.585	.69 [.18, 1.20]-.33 [-.87, .20].36 [-.43, 1.15].82 [.36, 1.29]-.37 [-.85, .10]-.22 [-.97, .52]	.009.219.364.001.122.551
Perceived workload			.06 [-.08, .19]	.414	.07 [-.07, .20]	.320
Team Support for Strengths use			-.05 [-.20, .10]	.520	-.03 -.18, .12]	.685
Interaction perceived workload*TSS					-.13 [-.32, .07]	.209
Teamlevel (intercept)	.21 [.13, .35]	.000	.21 [.12, .35]	.06	.21 [.13, .35]	.000
-2 Log Likelihood	1089.00		1024.30		1022.72	
Deviance change (Δ*χ*2 (df))	-		65.60 (2)**		1.58 (1)	
AIC	1115.00		1054.30		1054.72	
BIC	1167.80		1114.43		1118.86	

**significant at .01 level

*significant at .05 level

TSS = Team support for strengths use

**Fig 1:**
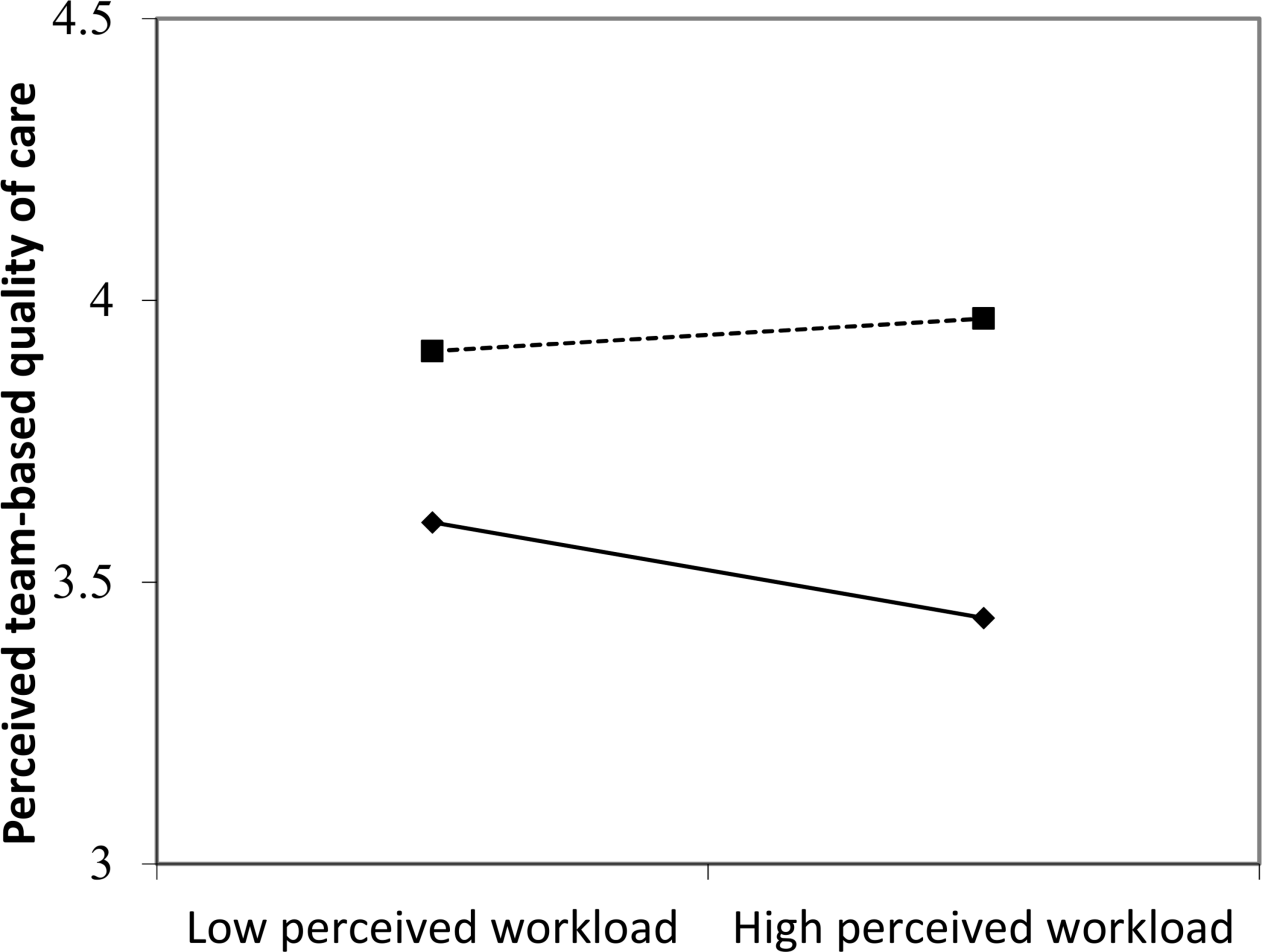
Relationship between perceived team-based workload, team support for strengths use, and perceived quality of care. –◆– Low team support for strength use. —■-—High team support for strength use.

Finally, the interaction between perceived workload and team support for strengths use was significant for perceived team-based quality of care (*B* = .14, *CI* = .03,.25), but not for fall incidents, medical errors, pressure ulcers, and aggression incidents (*B* = -.11, *CI* = -.29, .06; *B* = -.08, *CI* = -.26, .10; *B* = -.12, *CI* = -.28, .05; *B* = -.13, *CI* = -.32, .07, respectively). Fig 1 shows that perceived workload has a significant negative effect on perceived team-based quality of care when team support for strengths use is low (- 1 SD) (*B* = -.12, *p* = .02), but is unrelated to perceived team-based quality of care when team support for strengths use is high (+1 SD) (*B* = .04, *p* = .34). Based on the significance of the interaction term, Hypothesis 3a can be confirmed whereas Hypotheses 3b, 3c, 3d, and 3e remain unsupported.

## Discussion

### Key findings and discussion points

In contrast to previous studies [3–10] we did not find a significant relationship between perceived workload on the one hand and perceived team-based quality of care and patient safety on the other hand. This is in line with Myny et al. [56] who posit that so-called “non-direct patient care” factors such as hospital and ward characteristics, nursing team characteristics, characteristics of the individual nurse, patient and family characteristics may impact nursing workload and its effect on output.

Possibly, perceived workload is only associated with diminished quality and safety of care when job resources are lacking, as is also indicated by the interaction effect that we found between team support for strengths use and perceived workload. Our results point to strengths-based team support as a new job resource that might affect team performance and buffer the relationship between job demands and performance. Caregivers who perceive that their strengths are appreciated in their team and feel that they can capitalize on their strengths perceive higher quality of care provided by the team and this perception seems not to be affected by a heavy workload. Team support for strengths use may help caregivers to deal with high work load by alleviating stress levels, thereby reducing the risk that the quality of care is compromised. Our results demonstrate that leveraging the strengths of individual caregivers in the care team is directly associated with a higher perceived team-based quality of care and a lower perceived frequency of medication errors. However, perceived frequency of pressure ulcers, aggression, and fall incidents were not associated with the level of support given to caregivers’ strengths. Possibly, the general perception of quality of care provided by the team responds more quickly to team support for strengths use than more specific perceptions of the occurrence of adverse events. Another possible explanation is that these safety indicators depend more on the patients’ condition [57], such as their health, comorbidity, and self-efficacy, and are therefore less influenced by caregivers’ activities in comparison to medication errors. This may also explain why team support for strengths use did diminish the effect of perceived workload on perceived team-based quality of care, but did not diminish the effect of perceived workload on perceived frequency of adverse events (i.e., patient falls, medication errors, pressure ulcers, and aggression incidents).

### Implications for nursing management

Whereas providing skills training to caregivers often stimulates more concern for quality and safety of care [58], our results indicate that leveraging the unique strengths of individual caregivers in nursing home teams is also positively associated with perceived team-based quality of care and patient safety. Although personal strengths are trait-like characteristics that are energizing to the user, this does not mean that individuals are always aware of having them [59] or that strengths use is self-evident. Many people report not to make full use of their strengths at work [60, 61], due to their perceptions of normative demands at work, the appropriateness of strengths use, or their inclination to focus on fitting in at the expense of their authenticity [61, 62]. To create more awareness of the individual strengths present in a team, managers may use such instruments as Strengthsfinder [63], or the Values In Action Inventory of Strengths (VIA-IS) [24]. Alternatively, managers may conduct feedforward interviews (as opposed to feedback interviews), inviting employees to talk about moments when they felt energized at work, or caregivers may ask their colleagues in the team for feedback on when they are their ‘best self’ [64]. In a next step, managers and teams can discuss opportunities for team workers with complementary strengths to join forces, so that they can complement each other’s unique strengths.

### Limitations and implications for future research

The first limitation of this study is its cross-sectional design, which does not allow us to make causal inferences. Second, we used subjective measures of quality and safety of care because objective indicators at the team level were not available. So far, only few studies within the healthcare setting have combined subjective performance measures (e.g., satisfaction, perceived team-based quality of care, perceived performance, and processes) with objective performance measures (e.g., length of stay, nurse retention, hospitalization rates) [65]. Future research should attempt to apply a longitudinal design and to combine subjective and objective safety indicators in order to investigate two equally valuable angles on safety. Third, the relationship between team support for strengths use on the one hand and quality of care and patient safety on the other hand may be affected by common method bias because all constructs were rated by team members in one and the same questionnaire [66]. However, the results of a Harman’s one factor test in which all items that measured our three latent variables were loaded onto one common factor explained only 24.65% of the total variance, suggesting that common method bias does not affect our data [67]. Furthermore, the results from confirmatory factor analyses clearly indicated that loading all items on one common factor did not fit our data well, which suggests that common method bias may be limited. Also, the moderation effect that we found is unlikely to be biased by common source variance [68]. Nevertheless, future research should attempt to include data regarding quality of care and patient safety that is based on other sources, such as objective data, or ratings by team managers. A fourth limitation is that although our sample was fairly representative for Dutch nursing homes in terms of gender and age, it was not fully representative in terms of job title. Therefore, future studies should aim to collect randomly selected samples that are representative of the variety in nursing homes characteristics.

Future research could also explore the balance between tasks that need a uniform approach from all caregivers, and tasks that allow for individual differences and specialization. There is no doubt that caregivers need to be able to carry out a basic set of core tasks on the job, and must adhere to safety guidelines. However, for many tasks, such as dealing with aggressive or distressed patients, it is possible to allow for individual differences in the way the task is done, in line with the personal qualities of the caregiver [69]. Other tasks (e.g., drawing up schedules or communicating with relatives) may even be allocated to specific caregivers based on their individual strengths. The effect of team support for strengths use on the quality and safety of care is in line with the worldwide trend in specialization of the nursing profession, which often has a positive effect on nurses’ workload and job satisfaction, but is also fragmenting care delivery [70]. Future studies could further explore this balance between specialization based on individual strengths on the one hand, and uniformity on the other hand.

## Supporting information

S1 File Items team support for strengths use, workload, quality of care, patient safety, control variables. (DOCX)
