# Supplementary material for: Improving quality and safety of care in nursing homes by team support for strengths use: A survey study
Source: PLoS One. 2018 Jul 2;13(7):e0200065. doi: 10.1371/journal.pone.0200065 (PMC6028145; doi:10.1371/journal.pone.0200065)
Supplement: S1 File — (DOCX) [file pone.0200065.s001.docx]

**S1 File. Items team support for strengths use, workload, quality of care, patient safety, control variables**

|  | **Dutch** | **English** |
| --- | --- | --- |
| **Team support for strengths use** | | |
| 1 | In dit team word ik aangesproken op mijn sterke punten | In this team I am addressed for my strengths |
| 2 | In dit team heb ik de mogelijkheid om mijn kwaliteiten verder te ontwikkelen | In this team I have the opportunity to further develop my qualities |
| 3 | In dit team heb ik de mogelijkheid om te doen waar ik goed in ben | In this team I have the opportunity to do what I am good at |
| 4 | In dit team zijn mijn taken afgestemd op mijn sterke punten | In this team, my tasks are adjusted to suit my strengths |
| 5 | In dit team vullen de talenten van de teamleden elkaar goed aan | In this team the talents of the team members complement each other well |
| 6 | In dit team weten mijn collega’s wat mijn sterke punten zijn | My team members know what my strengths are |
| 7 | In dit team worden mijn sterke punten gewaardeerd | My strengths are appreciated in this team |
| **Perceived workload** | | |
| 8 | Binnen mijn team wordt onder tijdsdruk gewerkt | In my team, work is carried out under time pressure |
| 9 | Binnen mijn team wordt met pieken gewerkt | In my team, we work with peak times |
| 10 | Binnen mijn team moet te hard gewerkt worden | In my team, we need to work too hard |
| 11 | Binnen mijn team moet te veel werk verricht worden | In my team, too much work needs to be done |
| 12 | Binnen mijn team is te weinig tijd om het werk af te krijgen | In my team, there is too little time to finish the work |
| 13 | Binnen mijn team is het werktempo te hoog | In my team, the work pace is too high |
| 14 | Binnen mijn team is het werk mentaal veeleisend | In my team, the work is mentally demanding |
| 15 | Binnen mijn team is het werk te ingewikkeld | In my team, the work is too complicated |
| **Perceived team-based quality of care** | | |
| 16 | Door de goede samenwerking binnen ons team worden incidenten met cliënten voorkomen | The good cooperation within our team prevents incidents with patients |
| 17 | Ons team doet er alles aan om de fysieke veiligheid van teamleden te garanderen | Our team is committed to ensuring the physical safety of team members |
| 18 | Ons team heeft tevreden cliënten | Our teams has satisfied patients |
| 19 | Ons team levert goede kwaliteit | Our team delivers good quality of care |
| 20 | De manier waarop ons team werkt garandeert een goede kwaliteit van de hulpverlening | The way our team works guarantees a good quality of care |
| **Perceived patient safety** | | |
|  | Hoe vaak komen de volgende incidenten voor bij cliënten binnen uw team? | How frequently do the following incidents occur within your team? |
| 21 | valincidenten | Fall incidents |
| 22 | Medicatiefouten | Medication errors |
| 23 | Decubitus | Pressure ulcers |
| 24 | Agressie | Incidents of aggression |
| **Control variables** | | |
| 25 | Wat is uw geslacht? | What is your gender? |
| 26 | Wat is uw leeftijd? | What is your age? |
| 27 | Wat is de hoogste opleiding die u heeft afgerond? | What is your highest accomplished degree in education? |
| 28 | Wat is uw functie? | What is your job title? |
| 29 | In welk team bent u werkzaam? | To which team do you belong? |
| 30 | Uit hoeveel leden bestaat uw team? | How many members does your team consist of? |
| 31 | Hoe lang werkt u in dit team? (jaren) | How many years have you worked for this team? |
| 32 | Hoe lang werk u in deze organisatie? (jaren) | How many years have you worked for this organization? |
